# Molecular Determinants Involved in the Docking and Uptake of Tumor-Derived Extracellular Vesicles: Implications in Cancer

**DOI:** 10.3390/ijms25063449

**Published:** 2024-03-19

**Authors:** Irene Clares-Pedrero, Almudena Rocha-Mulero, Miguel Palma-Cobo, Beatriz Cardeñes, María Yáñez-Mó, Carlos Cabañas

**Affiliations:** 1 Tissue and Organ Homeostasis Program, Cell-Cell Communication Unit, Centro de Biología Molecular Severo Ochoa (CSIC-UAM), 28049 Madrid, Spain; irene.clares@cbm.csic.es (I.C.-P.); almudenarocham@gmail.com (A.R.-M.); miguel.palma@uam.es (M.P.-C.); bea_car@hotmail.com (B.C.); maria.yanez@cbm.csic.es (M.Y.-M.); 2 Departamento de Biología Molecular, Universidad Autónoma de Madrid, IUBM, 28049 Madrid, Spain; 3 Instituto de Investigación Sanitaria Hospital de la Princesa (IIS-IP), 28006 Madrid, Spain

**Keywords:** tumor-derived extracellular vesicles (TEVs), adhesion receptors, TEV docking, TEV uptake, integrins, tetraspanins, ICAM-1/CD54, ALCAM/CD166, CD44, ADAM17, ADAM10, CD9

## Abstract

Extracellular vesicles produced by tumor cells (TEVs) influence all stages of cancer development and spread, including tumorigenesis, cancer progression, and metastasis. TEVs can trigger profound phenotypic and functional changes in target cells through three main general mechanisms: (i) docking of TEVs on target cells and triggering of intra-cellular signaling; (ii) fusion of TEVs and target cell membranes with release of TEVs molecular cargo in the cytoplasm of recipient cell; and (iii) uptake of TEVs by recipient cells. Though the overall tumor-promoting effects of TEVs as well as the general mechanisms involved in TEVs interactions with, and uptake by, recipient cells are relatively well established, current knowledge about the molecular determinants that mediate the docking and uptake of tumor-derived EVs by specific target cells is still rather deficient. These molecular determinants dictate the cell and organ tropism of TEVs and ultimately control the specificity of TEVs-promoted metastases. Here, we will review current knowledge on selected specific molecules that mediate the tropism of TEVs towards specific target cells and organs, including the integrins, ICAM-1 Inter-Cellular Adhesion Molecule), ALCAM (Activated Leukocyte Cell Adhesion Molecule), CD44, the metalloproteinases ADAM17 (A Disintegrin And Metalloproteinase member 17) and ADAM10 (A Disintegrin And Metalloproteinase member 10), and the tetraspanin CD9.

## 1. Introduction

The term “Extracellular vesicles” (EVs) comprises a heterogeneous group of membrane-delimited structures released by almost all cell types both under normal and pathological conditions that play crucial intercellular communication roles in metazoan organisms. Classically, EVs were categorized into three main groups, exosomes (EXOs), microvesicles (MVs), and apoptotic bodies (APOs), according to their size and distinct mechanisms of biogenesis [1,2,3]; being exosomes, those EVs with an endocytic origin and a typical size of 40–150 nm in diameter; MVs (also termed ectosomes or microparticles) originating from direct budding of the plasma membrane and typically larger in size (100–1000 nm); and APOs deriving from the plasma membrane blebs formed in apoptotic cells, typically >1000 nm. However, due to their overlapping sizes and the lack of bona fide specific markers to define each type of EVs, the ISEV (International Society for Extracellular Vesicles) recommends the use of more general terms: small-EVs (S-EVs) and large-EVs (L-EVs) for vesicles of <200 nm and >200 nm, respectively [4].

A vast number of reports have shown that in most cancers, EVs produced by tumor cells (tumor-derived EVs or TEVs) influence all stages of cancer development and spread, including tumorigenesis, cancer progression, and metastasis. While both anti-metastatic and pro-metastatic effects have been attributed to TEVs, most reports indicate that the latter sort predominate in the vast majority of cancers (for excellent recent reviews, see [5,6,7]).

**Figure 1:**
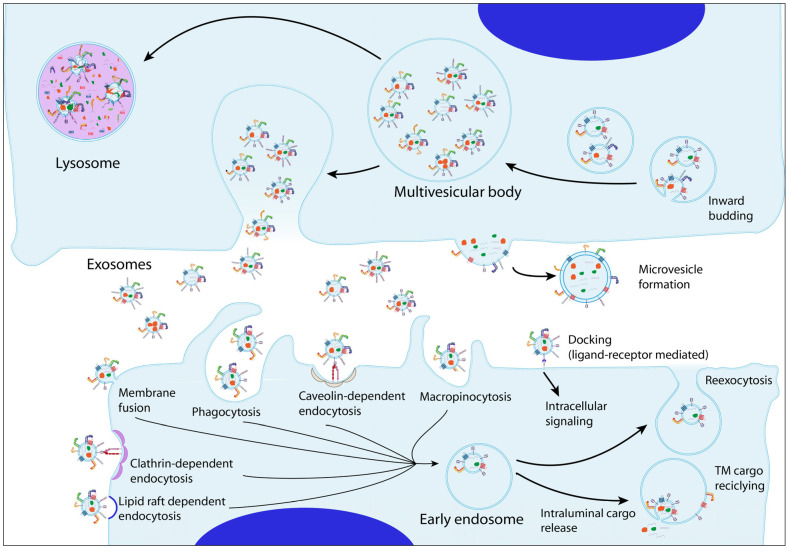
**EVs released from donor cells include microvesicles and exosomes.** These EVs carry a cargo of biomolecules (proteins, nucleic acids, and lipids) and can induce phenotypic and functional changes in target recipient cells through different general mechanisms. These include ligand-receptor mediated docking and subsequent intracellular signaling, membrane fusion, and EV uptake through macropinocytosis, phagocytosis, caveolin-, clathrin-, or lipid raft-dependent endocytosis.

TEVs can trigger profound phenotypic and functional changes in target cells through three main general mechanisms: (i) docking of TEVs on target cells and triggering of intracellular signaling; (ii) fusion of TEVs and target cell membranes with ensuing release of TEVs molecular cargo directly in the cytoplasm of recipient cell; and (iii) internalization of TEVs by recipient cells into endosomes (a process also termed “TEV uptake”), followed by subsequent delivery of their contents in the recipient cell’s cytoplasm upon back-fusion of the EVs with the limiting endosomal membranes [8,9]. These processes are not mutually exclusive and, in fact, docking of EVs on the target cell surface is frequently followed by their endocytic uptake and the release of their contents into the acceptor cell’s cytoplasm (Figure 1).

The docking of EVs on target cells occurs through interactions between molecular determinants—i.e., specific receptors and their cognate ligands or “counter-receptors”—that are expressed on the surface membrane of both EVs and recipient cells. These molecular determinants are typically proteins (including glycoproteins and proteoglycans), but lipids such as phosphatidylethanolamine, phosphatidylinositol or sphingolipids have also been described to play an important role in these interactions. Tetraspanins, integrins, members of the immunoglobulin superfamily (Ig-SF), proteoglycans, and lectins are amongst the proteins most frequently found to be involved in the docking of TEVs from a wide range of tumor sources, consistent with the well-known involvement of all these molecules in many different types of cell interactions and adhesive events. TEVs frequently express tumor antigens and mutated proteins on their surface that preferentially address them to certain cell lineages in specific target organs, contributing to the characteristic metastatic organotropism displayed by each type of cancer. In this regard, the interaction of ligands of the TNF-α (Tumor Necrosis Factor-α) family (TNF-α, FasL, TRAIL) with their respective receptors, as well as of PD-L1 (Programmed Death Ligand-1) with its receptor PD1, have been shown to mediate the docking of EVs produced by different cancer cells. Other adhesion molecules, such as CD44, ICAM-1/CD54, ALCAM/CD166, and integrins, have also been reported to mediate the interactions of TEVs with target cells. In some cases, the docking of TEVs on target cells also depends on particular extracellular cell matrix (ECM) proteins, such as fibronectin or laminin, that are bound on the external surface of EVs through specific receptors (including integrins, CD44, heparan sulfate proteoglycans) and act as bridge molecules that are also recognized by specific receptors expressed on the surface of the recipient cells [10,11,12,13]. Upon docking, multiple signaling pathways have been reported to be triggered by TEVs in target cells, either by direct stimulation of cell surface signaling receptors upon ligand–receptor engagement, or through the delivery of signaling components (contained in the biomolecular cargo of TEVs) following their fusion with or uptake by target cells, involving cytosolic calcium signaling [14], and the activation of specific kinases, such as FAK (Focal Adhesion Kinase), JNK (c-Jun N-terminal Kinase) [15], and MAPK/Erk (Mitogen Activated Protein Kinase/Extracellular-signal-regulated kinase) [16,17], the Wnt-PCP (Wingless integrated) [18], and NKG2D (Natural Killer Group 2 member D) signaling pathways (reviewed in [19]).

In few instances, EVs can fuse with the plasma membrane of recipient cells, thus directly releasing their intraluminal content into the acceptor cell cytosol. Direct fusion of EVs with the recipient cell plasma membrane has been monitored by employing EVs labeled with self-quenched lipophilic fluorescent dyes, which upon fusion with the unlabeled plasma membrane become diluted and result in dequenching of the dye and concomitant increase of fluorescence [20]. Although direct EVs fusion has been observed with dendritic and cancer cells [21,22], this process has not been thoroughly studied and the precise mechanisms that govern the fusion of EVs and target cell membranes remain incompletely characterized. They are suspected, however, to be similar to the processes employed by some enveloped viruses [23,24], where the fusion process proceeds through the formation of several intermediate structures (hemifusion stalk, hemifusion diaphragm, fusion pore) that are stabilized by protein scaffolds and requires specific fusogenic proteins [23,25].

Endocytosis of EVs by recipient cells is frequently referred to in the literature with the terms “EV internalization” or “EV uptake”. EVs can be internalized through different mechanisms, including clathrin- or caveolin-dependent endocytosis, lipid raft-mediated endocytosis, macropinocytosis and phagocytosis (reviewed in [8]). The involvement of any or a combination of these mechanisms in the process of EVs internalization by target cells has been inferred from the use of a wide range of inhibitors that block specific endocytosis pathways [8,26]. Internalization of EVs is an energy-dependent process that also requires a functioning cytoskeleton [8,26]. While many cell types seem to be capable of employing clathrin-, caveolin-, or lipid raft-mediated mechanisms to internalize EVs, macropinocytosis and phagocytosis are more restricted to specialized immune cells termed “professional phagocytes” such as macrophages, granulocytes, and dendritic cells, although some cell types, such as fibroblasts, epithelial, and endothelial cells can also accomplish phagocytosis with low efficiency. Phagocytosis requires specialized opsonic and non-opsonic phagocytic receptors, including lectins, specific integrins, and Fc-receptors [27,28].

Through the transfer of their biomolecular cargo of proteins, lipids and nucleic acids (mRNAs, miRNAs, and other non-coding RNAs) from the producing/donor tumor cells to a variety of recipient/target cells, TEVs have been shown to promote tumor microenvironment remodeling, angiogenesis, invasion, metastasis, and drug resistance in many different cancers, as recently reviewed in [6,7,29,30,31]. A plethora of proteomic, transcriptomic, and lipidomic analyses have focused on the identification of the specific molecules contained in TEVs that are responsible for the phenotypical and functional changes in recipient cells that ultimately result in cancer development and promotion. For instance, distinct miRNAs in TEVs have been identified as responsible for changes in target endothelial cells, both in the tumor microenvironment (TME) and at distant sites, resulting in increased vascular leakiness and angiogenesis, thus favoring cancer promotion [32,33]. Similarly, specific miRNAs contained in TEVs are responsible for the differentiation of different TME cells into CAFs (Cancer-Associated Fibroblasts), which in turn further promote cancer progression ([7,32,34,35]).

Although the overall tumor-promoting effects of TEVs as well as the general mechanisms involved in TEVs interactions with and uptake by recipient cells have been relatively well established and excellently covered in several comprehensive review articles [1,8,9,36,37], much lesser is currently known on the molecular determinants that mediate the docking and uptake of tumor-derived EVs by specific target cells. These molecular determinants dictate the cell and organ tropism of TEVs and ultimately control the specificity of TEVs-promoted metastases. Here, we will focus on reviewing current knowledge on selected specific molecules that have been reported to be involved in regulating the tropism of TEVs towards specific target cells and organs, including the integrins, ICAM-1, ALCAM, CD44, metalloproteinases ADAM17 and ADAM10, and the tetraspanin CD9.

## 2. Integrins in the Docking and Uptake of TEVs

Integrins are crucial cell adhesion receptors that mediate cell–cell, cell–ECM, and cell–pathogen adhesion phenomena, through interaction with their specific ligands. In addition to their adhesive function, integrins are also important cell signaling receptors that work in a bidirectional manner, through inside–out and outside–in signal transduction mechanisms [38,39,40]. Structurally, integrins are heterodimeric proteins formed by the non-covalent association of an α polypeptide subunit and a β polypeptide subunit. In vertebrates, the pairing of 14 different α subunits with 8 β subunits generates 24 different integrins. Integrins were originally categorized into several subfamilies (termed β1-, β2-, β3-integrins), according to the β subunit that is shared by different α subunits. An in-depth coverage of individual integrins expression and functions and description of their ligands is out of the scope of this review, but readers are referred to excellent reviews covering these aspects [40,41,42].

**Figure 2:**
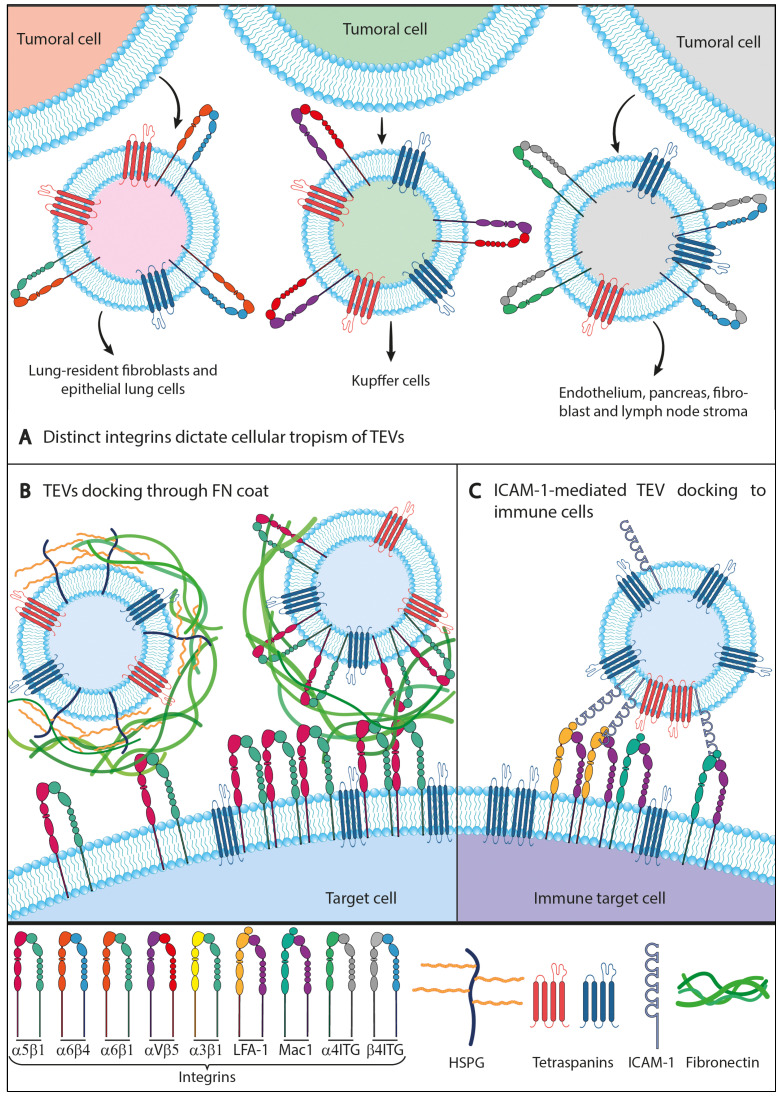
**Differential integrin expression can mediate TEVs docking, uptake, and tropism.** The presence of integrins on TEVs surface can guide their tropism or mediate their interaction and/or uptake with different target cells via ligand interaction. (**A**) Differential integrin expression profiles on TEVs surface can determine tissue organotropism. Integrins α6β1 and α6β4 on TEVs surface mediate lung-tropic metastasis; αVB5 mediates liver-tropic metastasis and the presence of α4 and/or β4 integrins usually correlates with TEV selectivity to endothelium, pancreas fibroblasts or lymph node stroma. (**B**) Some TEVs present a fibronectin coat on their surface when α5β1 integrins or Heparansulfate Proteoglicans (HSPGs) are present on their membranes. This fibronectin coat on TEVs mediates their docking to target cells via interaction with α5β1 integrins on the surface of target cells. (**C**) The presence of β2 integrins, such as LFA-1 or Mac-1, on the surface of immune target cells facilitates the docking of ICAM-1-surface-presenting TEVs.

Integrins (together with tetraspanins) are amongst the most characteristic and abundant membrane proteins on different types of EVs, including TEVs [43], and several studies have reported both direct and indirect roles for integrins in guiding the tropism and the specific interactions of these vesicles with their target cells. In a seminal article, the group of David Lyden elegantly showed that the specific subset of integrins present on the surface of TEVs produced by different types of cancer cells determines the organotropism of their metastases [44]. These authors found that integrins α6β4 and α6β1 on EVs were associated with lung metastases, while EVs carrying integrin αVβ5 were associated with liver metastases. These distinct integrins on TEVs dictate their selective uptake by certain resident cells in the destination organs, namely fibroblasts and epithelial cells in the lung, Kupffer cells in the liver, and endothelial cells in the brain, thus preparing the pre-metastatic niche in these distant organs. TEVs integrins triggered specific signaling pathways in target cells, including the activation of Src kinase and upregulation of pro-migratory and pro-inflammatory S100 molecules, which influenced the expression of genes involved in facilitating tumor metastases. Lyden’s group further showed that targeting the integrins α6β4 and αvβ5 with blocking reagents decreased EV uptake, as well as lung and liver metastasis, respectively, thus hinting at novel potential therapeutic avenues in cancer. Furthermore, based on clinical data, these authors proposed that EV integrins could be used to predict organ-specific metastases [7,44] (Figure 2A).

Likewise, using an innovative CRISPR-Cas9-based reporter system for single-cell detection of extracellular vesicle-mediated functional transfer of RNA, De Jong et al. found that β1 integrin (CD29) was a crucial molecule in EV-mediated RNA delivery to target cells [45].

The group of Margot Zöller has also demonstrated that the presence of distinct integrins that associate preferentially with the tetraspanin Tspan8, such as those containing the α4 or β4 chains, suffices to dictate the selectivity of TEVs towards endothelial, pancreas, fibroblasts, or lymph node stroma cells [46]. Through biochemical pull-down experiments and proteomics analyses, these authors also identified some of the ligands/counter-receptors present in the recipient cells which might be responsible for target cell selection. These ligands include the adhesion molecules MFG-E8/Lactadherin, Gal3bp, CD49e/integrin α5, CD54/ICAM1, CD106/VCAM1, CD56/NCAM, and CD44, which underscores the crucial role played by the interactions between specific tetraspanin-associated integrins and their cell-adhesion ligands/counter-receptors in dictating the target selectivity of TEVs.

Carney et al. reported that TEVs produced by SKOV-3 ovarian cancer cells express the integrin α3β1 and that this molecule is crucial for the uptake of these TEVs by other cancer cells. By screening focused combinatorial libraries of peptide or peptidomimetic molecules, these authors found peptide ligands highly specific for integrin α3β1. Specific targeting of this integrin on EVs with the cyclic nonapeptide LXY30 enabled the differentiation of cancer-associated EVs from non-cancer EVs and reduced the uptake of TEVs by target cells, pointing to novel diagnostic and therapeutic opportunities in ovarian cancer [47].

Different groups have described that the surface of TEVs from different cancer sources is coated with fibronectin (FN) and that this protein mediates the interactions of TEVs with specific FN receptors expressed on the surface of target cells. Reports indicate that this FN coating is required for the functional effects exerted by TEVs in target cells, such as guiding directional cell movement through tissues or promoting cell invasion [11,12,13]. Interestingly, FN has been shown to be bound on the surface of TEVs through different molecular entities, including the integrin α5β1 [11,13] and heparan sulfate proteoglycans [12] (Figure 2B). Although this FN coating may represent a relatively common phenomenon in TEVs produced by different types of cancer cells, our group has reported recently that in some instances TEVs do not display FN on their surface, as it is the case for TEVs produced by the human colorectal carcinoma Colo-320 cells [48], suggesting that in addition to FN, other molecular interactions can be also employed by TEVs to guide their targeting to recipient cells. Indeed, it has been shown both in vitro and in vivo that EVs can adsorb different proteins during their biogenesis within cells and from the extracellular fluids in which they originate and circulate (interstitial fluid, blood, urine, ascites, cell culture medium, etc.), thus forming a “protein corona” coat on their surface [49,50,51]. Both the intracellularly and extracellularly formed types of protein coronas may be relevant for EVs function [51]. Interestingly, Liam-Or et al. have very recently reported that the composition of the protein corona that wraps EVs dictates crucial properties of these vesicles, including their in vivo distribution as well as their targeting and uptake by specific recipient cells [52].

## 3. Roles of Integrin Ligand ICAM-1 (CD54) in the Docking and Uptake of TEVs

ICAM-1 (“InterCellular Adhesion Molecule-1”) or CD54 is a cell adhesion molecule of crucial importance during the extravasation of leukocytes at inflammation sites and in the formation and stabilization of immune synapses required for T cell-mediated immune responses [53,54,55]. Structurally, ICAM-1 is a type-I transmembrane protein that belongs to the immunoglobulin protein superfamily (IgSF), and comprises five extracellular immunoglobulin domains (D1–D5), one transmembrane domain, and a short cytoplasmic tail that links this molecule with the actin cytoskeleton [54,56]. ICAM-1/CD54 is constitutively expressed at relatively low levels on the endothelium, leukocytes, and many other cell types (including cancer cells), but its expression is dramatically increased by multiple inflammatory stimuli, including TNFα, IFN-γ, and IL-1 (reviewed in [57,58,59,60]). ICAM-1/CD54 is recognized primarily by integrin αLβ2 (also known as CD11a/CD18 or LFA-1 “Leukocyte Function-associated Antigen-1”) but is also known to be a ligand of integrin αMβ2 (CD11b/CD18 or Mac-1) and probably also of αXβ2 (CD11c/CD18 or gp150-95) [57,61,62], although in the latter case, it is not clear whether binding of ICAM-1 to integrin αXβ2 takes place directly or through fibrinogen, which would act as a bridge molecule between them [57]. Interestingly, integrins αLβ2, αMβ2, and αXβ2 share the common β2 subunit (CD18), are structurally closely related, and their expression is strictly restricted to leukocytes, although their expression on the different leukocyte subsets differs greatly amongst them [63,64,65,66]. While αLβ2 is expressed on virtually all types of leukocytes (although predominates in lymphocytes), the expression of αMβ2 and αXβ2 is much more restricted to myeloid cells, including granulocytes, monocytes, and macrophages, with αMβ2 predominating in neutrophils and αXβ2 in dendritic cells [62,67].

The participation of ICAM-1 (CD54) and its receptor αLβ2 in the docking/uptake of EVs by dendritic cells (DCs) was first reported by Morelli et al. in 2004, using blocking mAbs against these molecules [68]. Interestingly, these authors showed that EV tetraspanins CD9 and CD81 also participate in the docking and uptake of these vesicles by DCs. These findings were later confirmed by Zech et al., in the uptake of EVs produced by rat pancreatic adenocarcinoma BSp73ASML (ASML) tumor cells [69]. Through proteomic analyses, Rao et al. have found a substantial increase in ICAM-1 (CD54) expression in EVs derived from esophageal cancer tissue. These authors subsequently validated this finding with exosomes from plasma samples of esophageal cancer patients and proposed that EV CD54 could be a potential diagnostic marker for this type of cancer [70]. Li et al. have identified ICAM-1 on EVs produced by human prostate cancer cells as the key molecule that augmented the aggressiveness of other target prostate cancer cells in terms of migration and invasion capabilities. These authors established cell–cell communication via EV ICAM-1 as a novel mechanism by which the proto-oncogene RelB promotes prostate cancer progression [71].

Linton et al. demonstrated that the ICAM-1-mediated docking and uptake of human pancreatic ductal adenocarcinoma cells-derived EVs by macrophage-like THP-1 cells [72] enhanced their polarization towards an M2 immuno-suppressive and pro-tumoral phenotype, with was accompanied by enhanced secretion of soluble pro-tumoral bioactive molecules including VEGF, MCP-1, IL-6, IL-1β, MMP-9, and TNFα. Segura et al. showed by proteomic and biochemical analyses that EVs produced by mature DCs (“mature EVs”) are greatly enriched in ICAM-1, compared to EVs produced by immature DCs [73]. Furthermore, these authors also showed that ICAM-1 on mature EVs is required for naïve T cell priming, highlighting the relevance of this EV adhesion molecule in the induction of T cell-dependent immune responses. Along the same lines, ICAM-1 was detected on EVs produced by glioma cells, and DCs loaded with these EVs were able to activate T cells to become CTLs (cytotoxic T lymphocytes) that displayed vigorous cytotoxic activity against glioma cells [74]. A well-established pro-tumoral role of TEVs involves immune evasion through the inhibition of proliferation and cytotoxic capacity of CD8+ T cells [75]. In this regard, Zhang et al. have recently shown that ICAM-1 is crucial for this role of TEVs, as the adhesion of EV ICAM-1 to integrin LFA-1 (CD11a/CD18 or αLβ2) on CD8+ T cell is a prerequisite for subsequent interaction of EV PD-L1 with its receptor PD1 on T cells and for induction of immune suppression [76] (Figure 2C).

## 4. Roles of ALCAM-1 (CD166) in the Docking and Uptake of TEVs

ALCAM (“Activated Leukocyte Cell Adhesion Molecule”) or CD166 is another member of the immunoglobulin superfamily of cell adhesion molecules (IgSF-CAMs) that mediates cell–cell adhesion phenomena either through homophilic interactions between ALCAM molecules (ALCAM–ALCAM) on opposing cells or through heterophilic interactions with its ligand/counter-receptor CD6 (ALCAM–CD6). ALCAM is found in many tissues and cell types, but its expression seems to be restricted to specific subsets of cells involved in dynamic growth and migration processes [77,78,79,80]. ALCAM-mediated cell adhesion is relevant in different physiological phenomena, including collective cell migration, neuronal development, leukocyte extravasation, stabilization of the immunological synapses, T cell activation and proliferation, as well as in pathological settings including multiple sclerosis, autoimmune encephalomyelitis and tumor progression, invasion, metastasis and recurrence [55,80,81,82,83,84]. Furthermore, ALCAM expression has been proposed as a valuable prognostic marker in several types of cancer [81]. ALCAM/CD166 is a well-known substrate of the ADAM17 sheddase [85,86,87,88], which can shed its ectodomain from the cell surface as a 96 kDa soluble form (sALCAM) [82]. sALCAM has been proposed as a potential prognostic biomarker in several cancers [89], including epithelial ovarian cancer (EOC) [90], thyroid carcinoma [91], breast [92] and gastric cancer [93].

Several groups have reported that ALCAM/CD166 is expressed in TEVs produced by different types of cancer cells. Carbotti et al. detected the full-length transmembrane form (110 kDa) of ALCAM, but not the sALCAM (96 kDa), in EVs isolated from malignant ascites of ovarian cancer patients and from the conditioned media of cultured ovarian cancer cell lines [90]. Cardeñes et al. have recently reported that ALCAM/CD166 is involved in the docking of ovarian (OvC) and colorectal (CRC) cancer-derived TEVs and in their subsequent uptake by recipient target cells. Our group suggested that the identification of ALCAM/CD166 as a molecule mediating the docking and uptake of these TEVs could be potentially exploited to block the peritoneal metastasis cascade promoted by TEVs in CRC and OvC patients [94].

## 5. Roles of CD44 in the Docking and Uptake of TEVs

CD44 is a single-spanning type-I transmembrane glycoprotein endowed with both cell adhesion and signaling capacities. This molecule is abundantly expressed in embryonic stem cells and in many cell types in normal tissues. Remarkably, the expression of CD44 is highly upregulated in various cancers and this molecule is recognized as a molecular marker of cancer stem cells (CSCs). CD44 has been shown to affect numerous processes involved in cancer, including stemness, proliferation, invasion, metastasis, and drug resistance (for recent reviews, see [95,96,97]).

Distinct isoforms of CD44 are expressed on different cells as a result of alternative RNA splicing mechanisms. In humans, the gene coding for CD44 contains 20 exons, of which exons 1–5 and 16–20 are expressed by the “standard” or “non-variant” (also termed “hematopoietic”) isoform of CD44 (CD44s) from which exons 1–5 and 16–17 code for the extracellular domain, exon 18 for the transmembrane domain, and exon 19 or 20 for the intracytoplasmic tail. Inclusion of one or several exons from 6 to 15 in the mRNA gives rise to the different CD44 variant isoforms, all of which contain an additional variable polypeptide stretch in the juxtamembrane or “stem” region of the extracellular domain. For instance, the variant form CD44v6 contains exon 11 (i.e., the 6th exon after exon 5), while the variant form CD44v3-10 incorporates exons 8–15 (i.e., exons 3rd to 10th after exon 5). Distinct CD44v isoforms are expressed by different tumor cells and, therefore, may be used as markers of tumor progression and prognosis in particular cancers (reviewed in [95,96,97]. The expression of CD44s and CD44v isoforms is regulated in cancer cells at transcriptional and post-transcriptional levels. In this regard, multiple transcriptional repressors and activators have been identified to regulate CD44 promoter activity. Additionally, CD44 expression in cells is also regulated by epigenetic mechanisms and miRNAs (reviewed in [95]).

The complexity of the CD44 protein is increased by post-translational glycosylation of isoforms with O-glycans, N-glycans, and addition of glycosaminoglycans. Due to the modification of some forms of CD44 with glycosaminoglycans, such as chondroitin sulphate and heparan sulphate, some CD44 molecules are indeed bona fide proteoglycans [97,98,99,100]. Moreover, due to high variation in the level of glycosylation, the molecular weight of CD44 can be increased from 37 kDa (basic standard isoform) to 80–100 kDa, and can even surpass 200 kDa in some isoforms [97,100].

Still further adding complexity to the system, CD44 can also be detected as a soluble molecule (sCD44) in body fluids such as serum, lymph, arthritic synovial fluid, and bronchoalveolar lavage [101]. sCD44 can be generated either by secretion of a protein form translated from an alternatively spliced mRNA that lacks the transmembrane and intracytoplasmic domains, or by ectodomain shedding from the cell surface via proteolytic cleavage of transmembrane isoforms. CD44 ectodomain shedding can be executed by different metalloproteinases that belong to the MT-MMP [102] and ADAM [103] families. Interestingly, increased plasma levels of sCD44 have been associated with malignant disease and immune activation [104,105,106]. Amongst the ADAMs metalloproteinases, ADAM10 has been shown to be responsible for the shedding of sCD44 stimulated by Ca^2+^ influx, while the shedding of CD44 stimulated by activation of PKC and Rac seems to depend on ADAM17 [103,107,108,109].

The extracellular domain of transmembrane CD44 contains the interacting sites for binding of extracellular ligands, while the intracellular tail allows for interactions with several signaling and cytoskeletal proteins [97]. While several ligands and interacting proteins have been reported to bind specifically to CD44 (including, osteopontin, versican, serglycin, fibrin, MMP-14, MMP-9, MMP-2, FN, collagen, and integrins α6β4, α4β1, α5β1, αvβ3), hyaluronic acid (HA) is considered the main (and most extensively studied) ligand of CD44 [95,107,110,111,112]. CD44 ligands are either components of the ECM (such as FN, collagen, or HA) or adhesion receptors (such as integrins) expressed on the surface of other cells. Accumulating evidence supports that transmembrane CD44 molecules can exist in different states of activation, reflecting distinct capacities for ligand binding. On resting cells, CD44 generally shows low affinity for ligand binding (“inactive state”) but following cell stimulation/activation CD44 acquires the “active” state for binding HA with high affinity. The transition from the low-affinity state to the high-affinity state of CD44 can be induced by many different soluble stimuli, including cytokines and growth factors [113,114]. Activation of CD44 and subsequent HA binding brings about conformational changes in CD44 that facilitate the binding of adaptor molecules to its intracellular cytoplasmic tail which, in turn, trigger intracellular signaling that enhances cell adhesion, proliferation, migration, and metabolic shifts [95,115]. The signaling pathways that are activated following CD44-HA binding include Src, MAPK, and AKT/PI3K kinases, as well as Ras and Rho small GTPases (reviewed in [95,115]). Importantly, tumor cells tend to express CD44 constitutively in the active state with capacity for high-affinity HA binding.

CD44 is abundantly expressed on EVs derived from different tumor cells, and several reports have shown that the transfer of EV CD44 to distinct recipient cells plays a major role in promoting chemo resistance, tumor progression, and metastasis. In this regard, EVs produced by breast cancer cells treated with doxorubicin could spread resistance to this chemotherapeutic drug via the intercellular transfer of CD44 [116]. In ovarian cancer, EVs produced by tumor cells promoted peritoneal invasion through the transfer of CD44, which in turn triggered Mesothelial-to-Mesenchymal Transition (MMT) in peritoneal mesothelial cells (HPMCs) and increased secretion of MMP9 metalloproteinase [112]. Furthermore, the EV-mediated transfer of CD44 from high-metastatic ovarian cancer cells promoted migration and invasion of low-metastatic ovarian cancer cells, increasing their aggressiveness [117]. The precise mechanism by which CD44 on TEVs carries out these pro-tumoral and pro-metastatic effects in recipient cells has not been elucidated. Recently, EV CD44 has been shown to transmit metastatic capacity amongst gastric cancer cells through the triggering of signaling pathways that reprogram fatty acid oxidation in recipient cells [118]. A generalization of these findings would suggest that CD44 on TEVs could function as a crucial adhesion molecule directing these vesicles towards specific target cells that express the appropriate CD44 ligands or counter-receptors, such as HA or integrins. Upon TEVs docking or uptake by target cells, CD44 could also function as a trigger of signaling pathways impinging on cellular processes related to cancer progression and metastasis, such as proliferation, inhibition of apoptosis, migration or invasion.

EV CD44 has also been proposed to be instrumental in dictating the characteristic organotropism of cancer metastases. In a recent in vitro study by Mu et al. employing EVs produced by pancreatic cancer cells, EV CD44 was found to interact with integrin α6β4 promoting EV uptake by liver target cells [111]. The selective uptake of these EVs upregulated the expression of HGF, α-SMA, HA, and CD133, in liver cells, which are proposed to facilitate the generation of a pre-metastatic niche that ultimately would promote pancreatic tumor metastasis. Likewise, the study of Magoling et al. showed that depletion of CD44 on EVs derived from breast cancer cells significantly reduced their in vivo tumor delivery, which was evidenced by reduced tumor growth, thus highlighting the role of CD44 on TEVs surface in modulating their organotropism [119]. CD44 could also mediate the targeting of TEVs to recipient cells indirectly. It has been shown that CD44 on TEVs is involved in the assembly of a hyaluronate acid (HA) coat that surrounds the surface of these vesicles [115,120]. Another CD44 ligand, FN, has also been reported to coat TEVs and mediate recognition of these vesicles by integrin receptors (such as α5β1, α4β1, αvβ3) [11,13] or by heparan sulfate-bearing proteoglycans expressed on target cells [12].

## 6. The Dual Role of ADAM17 and ADAM10 Metalloproteinases in the Docking and Uptake of TEVs: Integrin Ligands and Surface Modifiers

ADAM (A Disintegrin And Metalloproteinase) proteins are a family of type-I transmembrane proteases with a modular structure encompassing (from N- to C-term) prodomain, catalytic, disintegrin, cysteine-rich, and EGF-like domains, followed by single transmembrane and cytoplasmic regions. Amongst the 21 ADAMs identified in the human genome, only 13 are actually proteolytically active while the rest lack the required Zn-binding motif in the catalytic domain [121,122,123]. Two closely related members of this family, ADAM10 and ADAM17, are crucial cell surface enzymes responsible for the cleavage and release of the ectodomains from a large variety of cell surface substrates, a process known as “ectodomain shedding”, which plays an essential role in numerous processes, including development, inflammation, and cancer [124,125]. ADAM10 and ADAM17 are atypical members of the ADAM family because the extracellular cysteine-rich and EGF-like domains are in these two members replaced by a unique membrane proximal domain (MPD), that is involved both in substrate recognition and regulation of their sheddase activity [86,126].

All ADAMs (including ADAM10 and ADAM17) contain a disintegrin domain in their extracellular region and can potentially act as a ligand for integrins. Some degree of selectivity has been reported for the interactions between the disintegrin domains of specific ADAMs and particular integrins [86,126,127], so that the integrin α5β1 has been shown to specifically recognize and bind the disintegrin domain of ADAM17 [128,129,130]. Of note, these specific α5β1-ADAM17 interactions can take place amongst molecules expressed on the same cell (cis interactions) and on different cells (trans interactions), with the latter type reported to support cell–cell adhesion phenomena [126,129]. Interestingly, cis interactions between integrin α5β1 and the disintegrin domain of ADAM17 have been shown to bring about the inhibition of both the adhesive capacity of the integrin (i.e., its ability to bind ligands) and the sheddase activity of ADAM17 because of steric hindrance that leads to decreased accessibility of its catalytic site for substrates [126,129]. On the contrary, dissociation of the α5β1-ADAM17 complex induces the activation of ADAM17 sheddase activity and enhances integrin-mediated cell adhesion.

On the one hand, our group has reported that the trans interaction between ADAM17 on TEVs and integrin α5β1 on target cells is involved in the binding and uptake of cancer-derived EVs, supporting a role for ADAM17 on TEVs as an integrin ligand and adhesion molecule [48].

On the other hand, several groups have reported that TEVs produced by different cancer types carry on their surface proteolytically active ADAM10 and ADAM17 [109,131,132]. Accordingly, these metalloproteinases could be (at least potentially) responsible for ectodomain release from multiple substrate proteins located on the TEVs membrane through a “cis shedding” (on the same membrane) mechanism. Of note, many biologically relevant substrate proteins for ADAM10 and ADAM17 are present on the surface of TEVs, including cytokines and growth factors (such as TNFα, TGFα, AREG, EREG, HB-EGF), growth factor receptors, and most relevantly, cell adhesion molecules such as CD44, L1-CAM, ICAM-1, VCAM-1, ALCAM, and β1-integrins, that are involved in the specific docking and uptake of TEVs. Therefore, through their potential to alter the balance between the EV surface (i.e., transmembrane) and the released (i.e., soluble) levels of these substrate proteins, ADAM10 and ADAM17 could dramatically influence the docking and uptake of these tumor-derived vesicles and hence their subsequent effects on target cells. Indeed, the constitutive and stimulated shedding of CD44 and L1-CAM adhesion molecules from TEVs have been shown to be predominantly mediated by ADAM10 and to a lesser extent also by ADAM17 [109].

Additionally, these metalloproteinases present on TEVs could also exert their proteolytic activity against substrate proteins expressed on recipient cells (“trans shedding” mechanism) as suggested in several reports. Groth et al. demonstrated that co-incubation of ADAM17-containing EVs with cells expressing the substrates TGFα and amphiregulin (AREG) led to increased shedding of both cytokines. This increased shedding was prevented when EVs were prepared from cells with shRNA-mediated ADAM17 knockdown, showing that ADAM17 (and in this case not ADAM10) was the sheddase responsible for the EV-mediated substrate release from target cells [131]. Hugendieck et al. have also shown that ADAM17 on TEVs isolated from the malignant ascites of ovarian cancer patients is responsible for the shedding of AREG from target tumor cells, representing another example of ADAM17-mediated “trans shedding” [132]. The released AREG could stimulate survival signaling in cancer cells by activating the receptor EGFR, revealing a potential chemoresistance mechanism mediated by TEVs in ovarian cancer cells [133].

**Figure 3:**
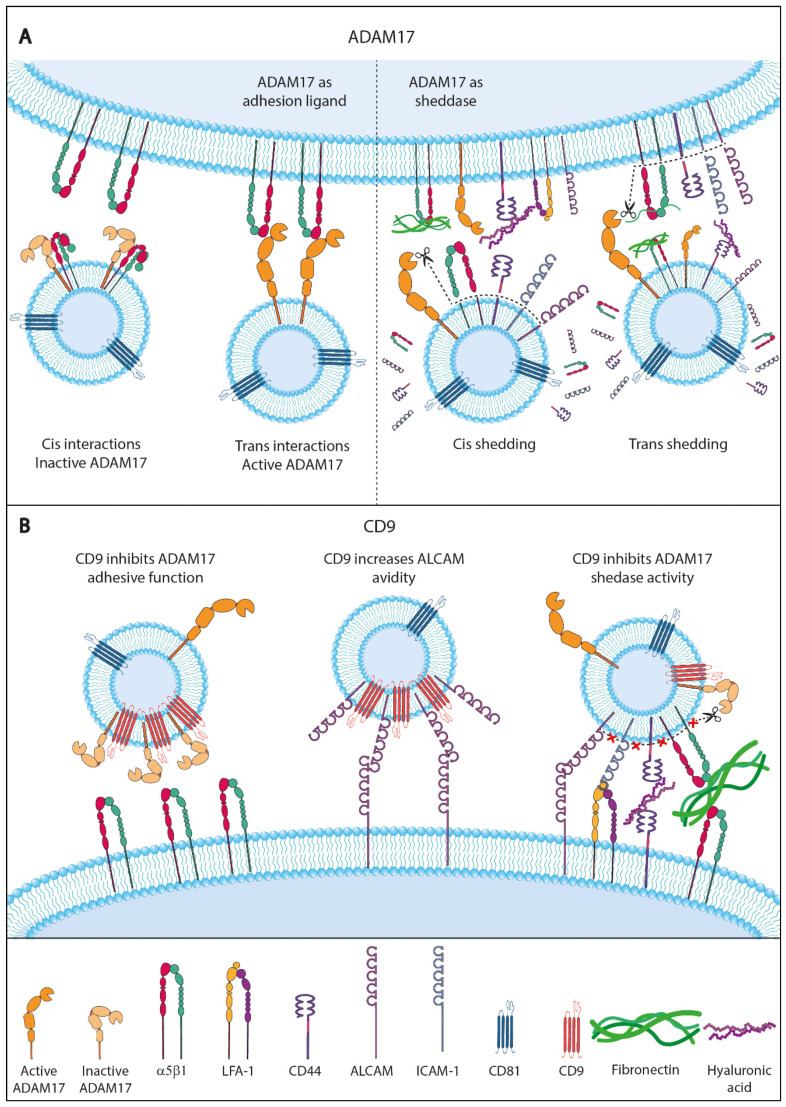
**Roles of ADAM17 and tetraspanin CD9 in the targeting and docking of TEVs.** (**A**) ADAM17 can influence the docking and targeting of TEVs to target cells through its dual role: (i) as an adhesion ligand that engages in *cis* and *trans* interactions with integrins; and (ii) as a sheddase that releases the ectodomains of its multiple transmembrane substrates, thus controlling the levels of many adhesive proteins on the surface of both TEVs and target cells. (**B**) Tetraspanin CD9 influences the activity of different adhesion receptors through a triple mechanism that involves the following: (i) augmented clustering of receptors on the cell and/or TEVs surface with resulting increase in their avidity; (ii) upregulation of the expression of ADAM17 transmembrane substrates on the surface of cells and TEVs due to CD9-mediated inhibition of ADAM17 sheddase activity; and (iii) inhibition of *trans* α5β1/ADAM17 adhesive interactions through imposition of unfavorable conformational changes on these molecules.

In sum, ADAM10 and ADAM17 can be important in the control of the docking and targeting of EVs to target cells through their dual role as (i) sheddases that control the levels of many adhesive proteins on the TEVs surface or target cells, and (ii) as an adhesion ligand themselves engaging in cis and trans interactions with integrins (Figure 3A).

## 7. Regulatory Roles of Tetraspanin CD9 in the Docking and Uptake of TEVs

Tetraspanins are abundantly expressed on the surface of most TEVs types (including tumor-derived exosomes) and, in fact, are widely used as key markers for the categorization of these vesicles [134]. Tetraspanins have been shown to be relevant in the biogenesis and cargo selection of EVs [135], being able to associate on membrane nanodomains with different adhesion receptors of the immunoglobulin and integrin families [136], including the integrin α5β1 [55,137,138].

In particular, the tetraspanin CD9 can regulate either positively or negatively the activity of the associated adhesion molecules [55,83,139,140,141,142]. Our group reported that the presence of CD9 on TEVs derived from human colorectal carcinoma Colo-320 cells decreased the ability of these vesicles to support cell adhesion as well as their uptake, which were both mediated through the interaction of TEV ADAM17 with cellular integrin α5β1. It was inferred that CD9 could impose a conformation on ADAM17 on TEVs that renders its disintegrin domain less accessible for binding by cellular integrin α5β1 [41]. Furthermore, expression of CD9 on both TEVs and recipient cells further reduced the adhesive capacity and the intake of these vesicles. Therefore, the association of CD9 with some adhesive molecules, namely ADAM17 on TEVs or integrin α5β1 on recipient cells, knocks down their adhesive function; indeed, these findings indicate that CD9 exerts a negative regulation on the docking and uptake of TEVs by recipient cells [48].

In other instances, on the contrary, CD9 enhances the functional capacity of associated adhesion molecules. This is the case for ALCAM-mediated homophilic (ALCAM–ALCAM) and heterophilic (ALCAM–CD6) cell–cell adhesion phenomena and signaling function, as reflected by the increased ALCAM-mediated cell adhesion and enhanced T cell migration, activation, and proliferation, observed upon ALCAM association with CD9 [55,83]. Whether CD9 could exert similar positive regulatory effects on the activity of associated adhesion proteins on the surface of TEVs has not been fully explored and deserves further research.

CD9 is also shown to associate directly with ADAM17 on the surface of different cell types (cis interactions), including leukocytes and cancer cells, and through this association exerts an inhibitory effect on ADAM17 sheddase activity against its substrates, including TNFα, ICAM-1 [83,143,144,145,146] or ALCAM [55,82,83].

Thus, CD9-mediated regulation of adhesion receptors function may be exerted through a triple mechanism that involves (i) the augmented clustering of receptors on the cell and/or TEVs surface and the resulting increase in their avidity; (ii) the upregulation of their expression on the cell and/or TEVs surface due to CD9-mediated inhibition of ADAM17 sheddase activity; or (iii) the inhibition of trans α5β1/ADAM17 adhesive interactions through imposition of unfavorable conformational changes (Figure 3B).

## 8. Concluding Remarks: Impact of TEV-Docking/Uptake Molecular Determinants in Cancer Therapy

Here, we have thoroughly reviewed different adhesion receptors exposed on the surface of tumor-derived EVs that have been shown to play a role in their docking and uptake. TEVs can trigger different outcomes in target cells impacting on metastases organotropism and immune system modulation. These effects may rely on firing intracellular signaling cascades through ligand–receptor interactions, release of active soluble ectodomains to the medium, or incorporation of bioactive TEV molecules such as miRNA or proteins into target cells. Thus, identification of the molecular determinants involved in these processes as well as a better characterization of their function and of the regulatory molecules and mechanisms that control them remains a daunting challenge in the field of cancer progression and metastasis. The huge therapeutic potential that could derive from interfering with the molecules that dictate the specific interactions and tropism of TEVs with selective target cells is easily envisioned and of utmost importance.

In the intricate landscape of cancer, molecules that serve both as biomarkers and key players in critical processes such as metastasis and chemotherapy resistance are of profound significance. Some progress has been made in utilizing these molecules as biomarkers (e.g., ALCAM, CD44, ICAM-1) [147,148]. In line with their role on TEV docking and uptake, these biomarkers could also allow improved prediction of metastasis organotropism, malignant traits such as invasiveness, and immunomodulatory capacity.

Despite their potential, clinical research on the direct therapeutic targeting of these molecules is still in its preliminary stages, with only a few translational studies identified. CD44 is the one for which more types of inhibitors have been developed and deeper clinical knowledge has been gained. Several studies targeting CD44 isoforms in different cancer types are reviewed by Chen et al., covering the use of antibodies, peptides, aptamers, pharmacological compounds, HA-mediated drug delivery to CD44 expressing cells or HA-CD44 interaction inhibitors [95]. For ADAM10 and ADAM17, pharmacological inhibitors have been developed and preclinical data showed promising results in different types of cancer [149], but in terms of clinical research, there seems to be only two clinical trials using the same ADAM10/17 inhibitor (INCB7839), one completed and the other one still ongoing (NCT02141451, NCT04295759). In the case of ICAM-1, there are abundant preclinical data with promising results, like the targeting of ICAM-1 using CAR-T cells in advanced thyroid cancer [150]. At clinical stages, alongside with B7.1 and LFA-3, ICAM-1 is one of the TRI-COM costimulatory molecules which have been used as T cell response boosters in vaccines against different types of cancer in combination with other treatments or antigens [151,152,153]. On account of tetraspanin CD9, there is also wide preclinical research about its role in cancer, but at the moment we can only highlight two approaches that are in clinical trials. One is a phase I clinical trial assessing the safety and efficacy of KBA1412 human monoclonal antibody targeting CD9 in patients with advanced solid tumors (NCT05501821), and the other one is the cell therapy approach based on CT0594CP, that combines CAR-T cells targeting BCMA and CD9 in patients with relapsed and/or refractory multiple myeloma or plasma cell leukemia (NCT05822037). However, none of the above studies centers its scope on the involvement of TEVs in the progression, spread, and resistance of the disease, where these molecular determinants may have a central role.

One of the crucial impacts of TEVs in the course of tumor progression is their capacity to modulate the phenotype and function of multiple types of immune cells (T cells, B cells, DCs, monocytes, neutrophils, MDSCs, and others), directing the immune response towards a protective immunosuppressive environment that promotes tumor growth and metastasis. Counteracting this effect could yield significant synergistic benefits when combined with currently available immunotherapies. The recent development of the tumor cell capture device M-TRAP, that targets peritoneal metastasis of ovarian cancer [154,155], the mentioned use of LXY30 to target TEVs from ovarian cancer [45], and recent results reported by Irep et al., showing that the treatment with inhibitors of exosome synthesis and trafficking (GW4869 and Nexinhib20) increases first-line chemotherapy treatment efficacy in vitro in small cell lung cancer (SCLC) [156], are excellent examples illustrating that, ultimately, TEVs themselves can also be employed in highly innovative approaches to help combat cancer treatment resistance as well as tumor metastases.

Additionally, a better understanding of the molecules dictating the docking and uptake of EVs can help improve the effectiveness of emerging exosome-based delivery strategies and technologies for cancer therapy developed by biotech companies, either as platforms to load and deliver therapeutic miRNAs and/or proteins or as immunomodulating agents [157,158,159,160,161,162,163].

This review aims to consolidate the existing knowledge on molecular docking/uptake determinants on TEVs and their collective role within the cancer context, providing a platform to propel further research towards clinical and therapeutic opportunities.
